# Histone Modifications Influence the Action of Snf2 Family Remodelling Enzymes by Different Mechanisms

**DOI:** 10.1016/j.jmb.2007.09.059

**Published:** 2007-11-30

**Authors:** Helder Ferreira, Andrew Flaus, Tom Owen-Hughes

**Affiliations:** Division of Gene Regulation and Expression, College of Life Sciences, University of Dundee, Dundee, DD1 5EH, UK

**Keywords:** Histone, Acetylation, Snf2, Nucleosome, Chromatin

## Abstract

Alteration of chromatin structure by chromatin modifying and remodelling activities is a key stage in the regulation of many nuclear processes. These activities are frequently interlinked, and many chromatin remodelling enzymes contain motifs that recognise modified histones. Here we adopt a peptide ligation strategy to generate specifically modified chromatin templates and used these to study the interaction of the Chd1, Isw2 and RSC remodelling complexes with differentially acetylated nucleosomes. Specific patterns of histone acetylation are found to alter the rate of chromatin remodelling in different ways. For example, histone H3 lysine 14 acetylation acts to increase recruitment of the RSC complex to nucleosomes. However, histone H4 tetra-acetylation alters the spectrum of remodelled products generated by increasing octamer transfer *in trans*. In contrast, histone H4 tetra-acetylation was also found to reduce the activity of the Chd1 and Isw2 remodelling enzymes by reducing catalytic turnover without affecting recruitment. These observations illustrate a range of different means by which modifications to histones can influence the action of remodelling enzymes.

## Introduction

Packaging DNA into nucleosomes and higher-order chromatin structures restricts access to the underlying genetic material. Thus, the manipulation of chromatin is a key step in many nuclear processes such as transcription, replication and repair. One of the means by which eukaryotes achieve this is through the use of ATP-dependent chromatin remodelling enzymes that non-covalently alter its structure. These enzymes are members of a diverse group of proteins named after the archetypal *Saccharomyces cerevisiae* Snf2 protein; the Snf2 family. Multiple members of this family of proteins are present in the sequenced genomes of eukaryotes of which the chromatin remodelling enzymes form distinct sub-groupings.^1^ Recent crystal structures of the catalytic domain of two Snf2 related proteins^2,3^ highlight structural similarities with the RecA domain found in a broad range of helicase related proteins. Snf2 proteins use the energy from ATP hydrolysis to alter DNA–protein interactions. However, unlike *bona fide* helicases, the action of chromatin remodelling enzymes is not generally associated with separation of DNA strands. Instead they act to catalyse dynamic transitions in chromatin structure. This can result in a variety of different outcomes. *In vitro*, chromatin remodelling enzymes have been shown to cause alterations to DNA accessibility, effects on DNA and chromatin topology, partial or complete removal or exchange of histones from nucleosomes and translational changes in the position of the intact histone octamer along DNA.^4^

How these proteins interact with chromatin and the mechanism by which chromatin remodelling is achieved remains unclear. However, in many cases it is apparent that histone tail domains are important regulators of chromatin remodelling. Genetically, there are links between histone tails and remodelling. Deletion of the H2B tail bypasses the need for the SWI/SNF complex in the regulation of several genes,^5^ and deletion of the H2A tail affects transcription of some SWI/SNF dependent genes.^6^ Importantly, post-translational modifications of histone tails affect chromatin remodelling. The recruitment and activity of the SWI/SNF complex *in vivo* is widely linked to that of the SAGA histone acetyltransferase complex.^7–9^ In addition, many chromatin remodelling complexes contain domains shown to bind modified amino acids, such as bromo- and chromodomains, PHD and WD40 domains.^10^ In these cases modifications have been proposed to act as epitopes that facilitate recruitment of proteins containing the appropriate recognition domains. For example, histone acetylation has been found to facilitate retention of SWI/SNF on nucleosome arrays^11^ in a way that is dependent on bromodomains within the Snf2 protein.^12^

The majority of the best characterised modifications occur within the non-globular histone tail domains which protrude from the core of the nucleosome. These histone tails constitute up to 30% by mass of histones, but are not visible in the crystal structures of nucleosomes due to their high intrinsic flexibility and have been thought to be largely unstructured.^13^ Their importance is highlighted by the fact that deletion of histone tails has wide spread effects on chromatin structure and gene regulation.^14–16^ Histone modifications have been correlated with a variety of chromatin states. On a genome-wide basis, histone H3 K4 tri-methylation and H3 acetylation are strongly correlated with active transcriptional start sites, phosphorylated H2A.X foci mark sites of DNA damage, and H3 K9 tri-methylation promotes the formation of heterochromatin *via* interaction with HP1.^10,17^ This suggests that histone post-translational modifications are an important means by which cells spatially and temporally regulate specific loci independently of bulk chromatin.

Rigorously studying the effect of histone tail modifications *in vitro* has been hampered by the difficulty in isolating histones with defined and homogeneous modifications. To circumvent this we have adopted an approach to chemically synthesise modified histones by means of native chemical ligation. This technology has previously been applied to generating chromatin with uniform histone N-terminal tail modification patterns.^18–20^ Using this approach we show that histone modifications can affect nucleosome remodelling *via* distinct pathways. Tetra-acetylation of histone H3 results in a modest increase in the rate of intrinsic nucleosome repositioning. The ATP-dependent remodelling enzyme RSC shows a striking preference for H3 but not H4 tetra-acetylated chromatin, remodelling this 16-fold faster than the control. By measuring the kinetic parameters of the remodelling reaction with a real-time ATPase assay, we show that this is due to a lower *K*_m_ value for H3 acetylated nucleosomes. In contrast, tetra-acetylation of histone H4 affects nucleosome remodelling by the Isw2 enzyme *via* altering the *K*_cat_ rather than the *K*_m_ of the ATPase reaction. We also show that the yeast Chd1 enzyme is dependent on the H4 tail for efficient nucleosome remodelling in a manner similar to Isw2. Remarkably, histone H4 tetra-acetylation affects the rate of RSC catalysed nucleosome transfer *in trans* but not nucleosome sliding *in cis*. Thus, nucleosome modifications can affect enzyme catalysed remodelling reactions by altering the outcome of the reaction, through allosteric effects on ATPase activity and by acting as binding epitopes for recruitment.

## Results

### Generation of modified chromatin

**Figure 1:**
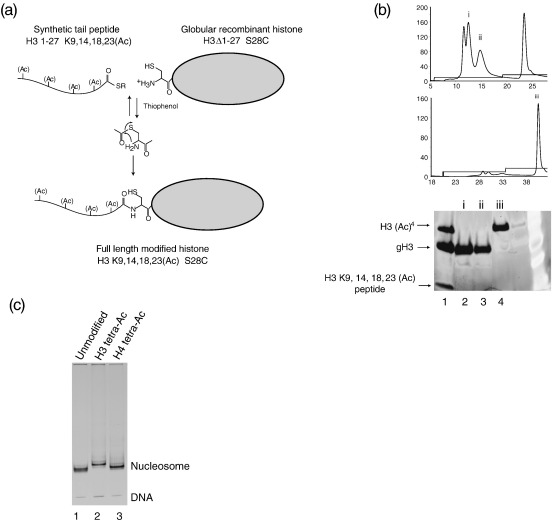
Generating acetylated histones and nucleosomes. (a) Modified histones were generated by the ligation of chemically synthesised peptide bearing a C-terminal thioester group to an N-terminally truncated histone bearing an N-terminal cysteine. In this case the peptide consisted of amino acid residues 1–27 of the *Xenopus laevis* H3 tail in which lysine residues 9, 14, 18 and 23 were acetylated and K27 synthesised as a thioester derivative. The histone was histone H3 in which amino acid residues 1–27 had been truncated and the new N-terminal residue mutated from serine to cysteine. The ligation reaction proceeds *via* an irreversible intramolecular rearrangement to produce a covalent native peptide bond without the introduction of unnatural chemical moieties. (b) Full-length acetylated histones were purified away from the reactants by two rounds of ion exchange chromatography using a step gradient. Lane 1 shows a representative reaction in which ligation had proceeded to around 40%. The top two panels show the traces from the first and second chromatography runs used to purify the ligated product from the unreacted material. The bottom panel shows SDS–PAGE of fractions taken form the positions (i, ii, and iii) indicated on the traces. Fraction iii, consists of over 95% ligated full length histone H3. (c) Acetylated histone octamers formed nucleosomes similar to those formed by unmodified histone octamers. Interestingly, the mobility of tetra-acetylated H3 nucleosomes during native PAGE was slightly reduced compared to control nucleosomes of the wild-type sequence; compare lanes 1 and 2 or histones containing cysteine point mutations (not shown).

Modified histones were generated by ligating a bacterially expressed *Xenopus laevis* globular histone containing an N-terminal cysteine and a tail peptide with the modification of choice synthesised chemically as a C-terminal thioester. The ligation was based on the protocol described by Dawson & Kent^21,22^ and used thiophenol as the catalyst (Figure 1(a)). A key requirement of the globular histone is that it has an uncapped N-terminal cysteine available for ligation. One strategy that has been used previously to ensure that the initiator methionine is removed is the introduction of a protease cleavage site just before the cysteine to expose it post-translationally.^20^ Mass spectrometry (Supplementary Data, Figure 1) and the work of other laboratories^19,23^ show that removal of the initiator methionine *in vivo* by *Escherichia coli* methionine aminopeptidases is efficient when cysteine is the adjacent amino acid. This alleviates the requirement for more complex systems for generation of N-terminal cysteine residues.

Polyacrylamide gel electrophoresis reveals that under the conditions used, ligation does not proceed to completion (Figure 1(b), lane 1). However, the full-length modified histone can be separated from the unreacted globular domain and tail peptide *via* multiple rounds of ion exchange chromatography to obtain material that was greater than 95% pure (Figure 1(b)). The overall efficiency of ligation and subsequent purification is approximately 20% (data not shown). When modified histones were combined with other core histones to form histone octamers and purified by size exclusion chromatography, they displayed identical elution profiles to unmodified octamers (data not shown). Modified histone octamers could be reconstituted onto DNA fragments to form nucleosomes. Interestingly, acetylation of H3 and to a lesser extent H4 reduced the migration of nucleosomes on native polyacrylamide gels (Figure 1(c)). This has been observed previously in the Bradbury laboratory using hyperacetylated histones purified from HeLa cells^24^ and suggests that linker DNA conformation may be affected. This altered electrophoretic migration is not due to the ligation procedure as nucleosomes containing methylated lysine residues did not show this behaviour (not shown).

### Lysine acetylation can alter intrinsic nucleosome mobility

**Figure 2:**
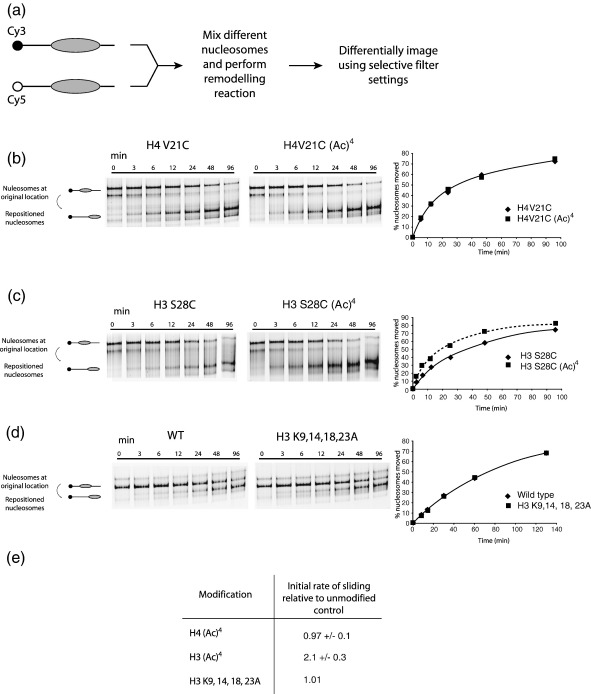
Effects of histone acetylation on intrinsic nucleosome mobility. (a) Outline of competitive remodelling assay used to accurately measure differences between nucleosomes. This setup has the advantage that the reaction times and conditions are exactly the same for two nucleosomes and avoids the possibility of observing effects due to different nucleosome reconstitution efficiencies. (b) Histone H4 tetra-acetylation does not alter the rate at which nucleosomes reposition thermally. Two pmol of H4 acetylated and H4 V21C unmodified nucleosomes assembled on 54A54 DNA were mixed and incubated at 47 °C for the specified amount of time. The images represent the Cy3 (H4 V21C) and Cy5 (H4 acetylated) scans of the same gel. The amount of remodelling is plotted to the right of the gels and a hyperbolic curve fitted to the data points. From this the initial rate of repositioning is calculated and the average of three independent repeats displayed in (e). (c) Tetra-acetylation of H3 results in a twofold increase in the rate of intrinsic nucleosome mobility. Reaction conditions are as for (b). (d) The increase in nucleosome mobility by H3 acetylation is not solely due to charge neutralisation. Two pmol of H3 wild-type and H3 in which the lysine residues at position 9, 14, 18 and 23 had been substituted to alanine were assembled on 54A18 DNA were mixed and incubated at 47 °C for the specified amounts of time. Substituting the lysine residues with alanine, which will reduce the basic charge of the tail, had no effect on nucleosome mobility. (e) Table indicating the initial rate of repositioning relative to control for the three constructs described above.

Although nucleosomes are stable with respect to dissociation, they can undergo a range of dynamic fluctuations in their structure. For example, following thermal incubation nucleosomes are frequently observed to redistribute to thermodynamically favoured locations.^25^ This movement of nucleosomes from one translational position on DNA to another can be followed using native PAGE.^26^ Acetylated and control nucleosomes were reconstituted onto differentially labelled fluorescent DNA, mixed, incubated thermally and run on a native gel (Figure 2(a)). The different nucleosomes can then be visualised separately using selective excitation and emission filters. From this the proportion of nucleosomes at the initial and final position can be measured at each time point. The rate at which nucleosomes accumulate at their destination can then be plotted and a hyperbolic curve fitted to the data enabling the initial rate of redistribution to be estimated (Figure 2(b)–(e)). This approach has proved more reliable at directly comparing the behaviour of different nucleosomes.^27^

Although native thiol ligation results in a normal peptide bond without the introduction of unusual chemical moieties, it requires the presence of a cysteine residue at the point of ligation. For this reason, the unmodified control nucleosomes used as a reference are point mutants containing a cysteine at the equivalent position to the modified histones. The sites selected for ligation involved substitution of cysteine for amino acids with similar overall dimensions (e.g serine to cysteine). None the less it was important to show that introduction of these mutations did not affect the behaviour of nucleosomes. Supplementary Data, Figure 2 shows a comparison of the thermal mobility of wild-type and cystein point mutant nucleosomes showing that the cystein mutations themselves have no discernable effect. The effects of the cysteine point mutations were also compared to wild-type histone sequences in other assays described below and in all cases no significant effect was detected (Supplementary Data, Figures 2, 3, 4; and data not shown). As a histone bearing the relevant cysteine point mutation is most closely matched to the product of a peptide ligation reaction, we have generally presented data using the point mutant as a control rather than the wild-type histone sequence. However, we are not aware of any cases where the choice of control affects the conclusions drawn.

H4 acetylated nucleosomes were observed to be repositioned at the same rate as control nucleosomes (Figure 2(b)). In contrast, nucleosomes containing acetylated H3 relocated twice as fast as the control (Figure 2(c) and (e)). To test to what extent this was due to the partial neutralisation of basic charge that occurs upon acetylation of lysine residues, a histone H3 construct with lysine to alanine mutations at the four acetylated positions (H3 K9, 14, 18, 23A) was compared to wild-type (Figure 2(d) and (e)). This nucleosome repositioned at the same rate as the control indicating that charge neutralisation is not the sole mechanism by which acetylation affects nucleosome mobility.

### The RSC complex preferentially repositions tetra-acetylated H3 but not H4 nucleosomes

**Figure 3:**
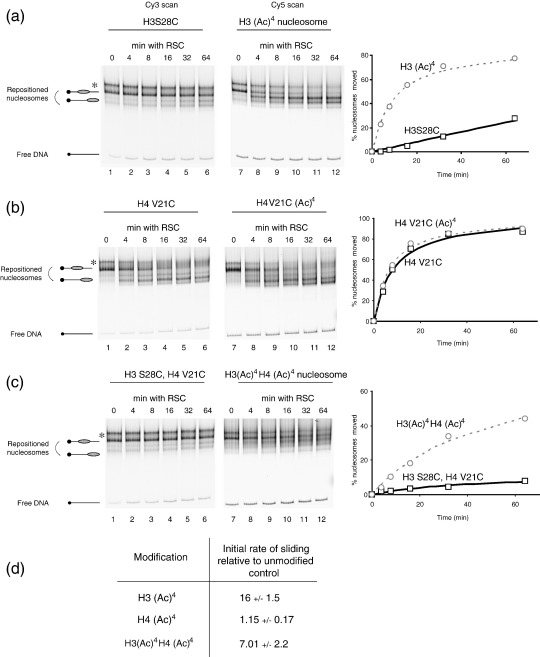
RSC preferentially repositions tetra-acetylated H3 nucleosomes. (a) 20 fmol of RSC were incubated with 1pmol unmodified S28C octamers assembled onto Cy3-labelled 54A18 DNA and 1pmol H3 tetra-acetylated nucleosomes on the same DNA labelled with Cy5 and incubated for the specified length of time at 30 °C in the presence of 1 mM ATP. RSC shows a dramatic preference for H3 tetra-acetylated nucleosomes: compare lanes 1–6 and 7–12; see also (d). On this fragment a proportion of nucleosomes are deposited at an alternative location, indicated by an asterisk (*), that has been characterised previously.^61^ Inclusion of nucleosomes deposited at this location had little effect on the calculated initial rate of sliding so they were excluded from quantitative analysis. (b) H4 tetra-acetylated nucleosomes, in contrast, are not repositioned faster by the RSC complex. (c) Nucleosomes that are both H3 and H4 tetra-acetylated are not repositioned any faster than H3 tetra-acetylated nucleosomes, confirming that H4 tetra-acetylation does not promote RSC catalysed repositioning. (d) Table indicating the average initial rate of repositioning relative to control and standard deviation from three independent experiments for the acetylated constructs described above.

RSC is an abundant and essential yeast chromatin remodelling complex that is closely related to SWI/SNF.^28^ A notable feature of the RSC complex is that it contains almost half of the known bromodomains in the *S. cerevisiae* genome. As bromodomains within other proteins have been found to recognise specific patterns of histone acetylation,^29,30^ a distinct possibility is that histone acetylation will influence the action of the RSC complex. To test this, the ability of RSC to remodel either H3 or H4 acetylated chromatin relative to unmodified control chromatin was assayed. RSC showed a dramatic preference for H3 (K9, 14, 18, 23) tetra-acetylated chromatin compared to an unmodified control (Figure 3(a)). From the initial rates of remodelling derived from the plotted data, this was calculated to be 16(±1.5)-fold faster than control (Figure 3(d)). When the rate of H4 (K5, 8, 12, 16) tetra-acetylated nucleosomes was measured, this was found to be indistinguishable from the rate of an unmodified control (Figure 3(b) and (d)).

It is also worth noting that although the rate at which the H3S28C control nucleosomes are shifted in Figure 3(a) is slower than the rate at which the H4V21C nucleosomes are shifted in Figure 3(b), this should not be interpreted as indicating that the S28C mutation reduces the rate at which nucleosomes are repositioned by RSC. The reason that S28C nucleosomes are moved slower is due to the preferential engagement of RSC with acetylated nucleosomes at the expense of non-acetylated nucleosomes in the reaction mixture, as they are essentially in competition with each other. Indeed, comparing the rate of acetylated nucleosomes to either wild-type or cysteine containing control nucleosomes shows the same effect (Supplementary Data, Figure 3) This illustrates the importance of only making direct comparisons between the two templates present in the same remodelling assay. In this case, our data show that H3 acetylation stimulates nucleosome slding whereas H4 acetylation does not. Thus, none of the seven bromodomains found within the RSC complex appear to interact with acetylated lysines within the H4 tail in a way that affects nucleosome sliding. This is consistent with the observation that the relative rate of remodelling of a H3 and H4 acetylated nucleosome is not faster than a nucleosome that is just acetylated on the H3 tail (Figure 3(c) and (d)).

### H3 tetra-acetylation influences remodelling by the RSC complex by affecting *K*_m_ but not *K*_cat_ of ATP hydrolysis and acts largely *via* H3 K14 acetylation

**Figure 4:**
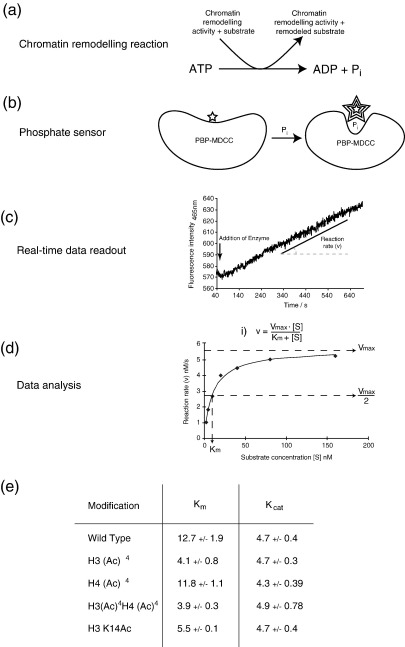
Determining the kinetic parameters of nucleosome remodelling by RSC with a fluorescent ATPase assay. Overview of ATPase assay. (a) Nucleosome remodelling generates the release of inorganic phosphate (Pi) as a result of ATP hydrolysis. (b) This level of Pi is detected by a fluorescently labelled phosphate binding protein (PBP–MDCC), whose fluorescence increases dramatically upon phosphate binding. (c) Chromatin remodelling is initiated by the addition of 0.3 nM RSC to different concentrations of 36W36 nucleosomes and the fluorescence intensity measured in real-time at 1 s intervals over approximately 10 min. (d) Kinetic parameters were calculated by non-linear fitting of the Michaelis–Menton equation to the plotted data. (e) *K*_m_ and *K*_cat_ of remodelling of different nucleosome substrates by RSC. H3 tetra-acetylated nucleosomes show lower *K*_m_ values without affecting *K*_cat_. H4 tetra-acetylation does not affect either parameter, consistent with data from Figure 3. Mono-acetylation at K14 of H3 significantly affects the *K*_m_ of remodelling, largely mimicking H3 tetra-acetylation. Although the *K*_m_ and *K*_cat_ shown above were calculated for wild-type histones no difference was detected for H3S28C nucleosomes (Supplementary Data, Figure 4).

The increased rate with which RSC repositions H3 acetylated nucleosome could be due to the modified lysine residues acting to recruit RSC or by allosterically affecting the remodelling reaction. To differentiate between these two options, the kinetic parameters of the ATP hydrolysis reaction were measured using a real-time fluorescent ATPase assay.^31^ The assay hinges on using a phosphate binding protein (PBP) labelled with a coumarin-based fluorescent dye, 7-diethylamino-3-((((2-maleimidyl)ethyl)amino)carbonyl)coumarin (MDCC), as a sensor for the amount of inorganic phosphate (Pi). On binding Pi, the labelled protein (MDCC-PBP) undergoes a shift in its emission wavelength coupled with a fivefold increase in fluorescence. When performed in a fluorimeter, this assay has the advantage of measuring ATP hydrolysis in real-time, from which kinetic parameters such as *K*_m_ and *V*_max_ are determined by non-linear fitting to the Michaelis–Menton equation (Figure 4(a)–(d)). We find that RSC has approximately threefold lower *K*_m_ (tighter binding) for H3 acetylated nucleosome compared to the unmodified control, without affecting the *K*_cat_ of ATP hydrolysis (Figure 4(e)). This is consistent with preferential recruitment of RSC to H3 acetylated chromatin and the preferential binding of RSC to H3 acetylated nucleosomes (Supplementary Data, Figure 5). In contrast, RSC does not preferentially bind H4 acetylated nucleosomes, as the *K*_m_ for these is the same as for the unmodified control (Figure 4(e)). Consistent with this, the *K*_m_ value of the doubly H3, H4 acetylated nucleosome is indistinguishable from that of the H3 acetylated nucleosome (Figure 4(e)).

Previous work from the Cairns laboratory has suggested the tandem bromodomains of the RSC4p subunit of the RSC complex interact with acetylated H3 K14.^32^ To test whether the single acetylation of lysine 14 is responsible for the dramatic effect of H3 (K9,14,18,23) tetra-acetylation on RSC activity, the singly acetylated nucleosome was generated and put through the same ATPase assay described above. From Figure 4(e) it can be seen that as for H3 tetra-acetylated nucleosomes, the *K*_cat_ value remains unchanged. In contrast, the *K*_m_ value was significantly lowered, almost to the levels of the tetra-acetylated construct, suggesting that acetylation at a single residue, K14, confers the majority of the effect observed upon tetra-acetylation of the H3 tail.

### The H4 tail and its acetylation influence remodelling by Isw2 by affecting the *K*_cat_ of ATP hydrolysis

**Figure 5:**
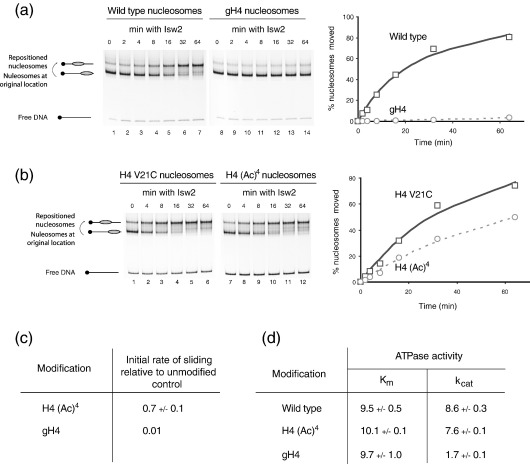
H4 tail regulates the catalytic activity of Isw2. (a) As Isw2 has been observed to move nucleosomes away from DNA ends, nucleosomes were assembled at a position close to a DNA end using the fragment 54A0. 3 fmol of Isw2 were incubated with 1pmol intact octamers assembled onto Cy3-labelled DNA and 1pmol octamers from which the first 19 amino acid residues of H4 had been deleted assembled on Cy5-labelled DNA for the times indicated at 30 °C in the presence of 1 mM ATP. gH4 nucleosomes were repositioned slower as shown in the graph. (b) In a similar comparison tetra-acetylated nucleosomes are repositioned slower than H4 V21C nucleosomes by Isw2. (c) Quantitative comparison of the effects of truncating and acetylating the H4 tail on Isw2 atpase activity. (d) Effects of H4 acetylation and truncation on the ATPase activity of Isw2. Reaction conditions are as for Figure 4 except using 0.2 nM Isw2 and different concentrations of 54W0 nucleosomes. Truncation of the H4 tail, and to a lesser extent histone acetylation, reduce the catalytic turnover of the enzyme without significantly affecting the *K*_m_.

Subfamilies of Snf2 family chromatin remodelling proteins contain different motifs capable of interacting with histones raising the possibility that they may be regulated in different ways by histone modifications. One of the defining characteristics of the Iswi subfamily of remodelling enzymes is that they require a particular epitope within the H4 tail for efficient remodelling activity.^33–36^ One of the lysine residues adjacent to this motif, H4 K16, can be acetylated, and there is evidence that this reduces the activity of ISWI containing remodelling complexes.^19,37^ When we compared the relative rate of mobilisation of H4 tetra-acetylated nucleosomes relative to an unmodified control by the yeast Isw2 enzyme, we found that the rate is approximately 1.5-fold slower (Figure 5(b) and (c)). This reduction, whilst significant, is not as severe as deletion of the H4 tail (Figure 5(a) and (c)). However, its biological importance is underlined in studies on flies, where defects in the genetic interaction between H4 K16 acetylation and ISWI result in large scale chromosomal abnormalities.^37^

Applying the same kinetic analysis performed previously to RSC shows that deletion or acetylation of the H4 tail lowers the turnover rate of the hydrolysis reaction (*K*_cat_) without affecting nucleosome binding (*K*_m_) (Figure 5(d)). We have confirmed this by performing gel shifts of either full length or gH4 nucleosomes with Isw2 and observe no difference in binding (Supplementary Data, Figure 6). This contrasts with the effect of histone acetylation on remodelling by the RSC complex and shows that histone modifications can affect chromatin-remodelling enzymes at different stages of the reaction cycle.

### The yeast Chd1 enzyme requires the H4 tail for efficient chromatin remodelling

**Figure 6:**
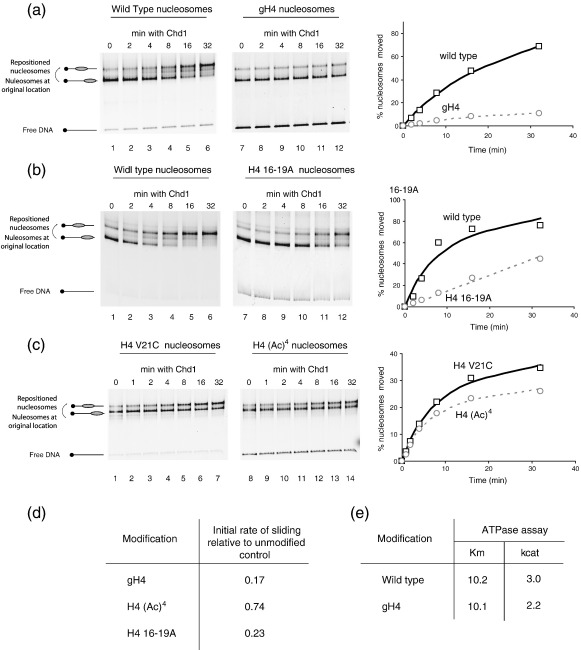
Chd1 requires the H4 tail for efficient nucleosome remodelling. (a) 40 fmol of Chd1 were incubated with 1pmol of wild- type octamers deposited on Cy3-labelled 54A0 DNA and 1pmol of octamers containing H4 with the first 19 amino acid residues deleted assembled on the same DNA labelled with Cy5 and incubated for the specified lengths of time at 30 °C in the presence of 1 mM ATP. Deletion of the H4 tail results in poor nucleosome remodelling by Chd.1 (b) Mutation of amino acid residues 16–19 to alanine significantly reduces the rate at which Chd1 repositions nucleosomes. (c) Tetra-acetylation of H4 causes nucleosomes to be repositioned at a slower rate by. (d) Quantification of the effect of alterations to histones on the initial rate of nucleosome repositioning by Chd1 shown in (a)–(c). (e) Effect of truncating the H4 tail on the ATPase activity of Chd1. The reaction conditions are as for Figure 4 except using 1 nM Chd1 and 54W0 nucleosomes initial rates of ATP hydrolysis were measured. A non-linear fit of these data to the Michaelis–Menton equation (with R2 confidence values above 0.98) allows *K*_m_ and *K*_cat_ to be determined for nucleosomes containing intact H4 and truncated H4. There is little effect on *K*_m_, but *K*_cat_ is significantly reduced, suggesting that the H4 tail is an allosteric effector for Chd1.

Chd1 belongs to a phylogenetic subfamily that is distinct from either of the previously studied enzymes^1^ and contains a chromodomain that has the potential to recognise histone modifications. To investigate the histone dependence of this enzyme, we first tested the effect of deleting individual histone tails. We found that Chd1 is unable to efficiently slide nucleosomes lacking the H4 amino terminal tail (approximately sixfold slower than control: Figure 6(a), compare lanes 1–6 with 7–12). This requirement for the H4 tail is similar to that seen with Iswi remodellers such as Isw2, although the magnitude of the effect is lower. Mutation of residues 16–19 to alanine caused a 4.4-fold reduction in Chd1 activity (Figure 6(b)). Although this effect is significant it does not fully match the effect of a H4 truncation raising the possibility that the amino acids recognised by Chd1 include residues in addition to those within the 16–19 region.

Acetylation of residues within the H4 tail also affected Chd1's nucleosome remodelling activity. Indeed, when this was tested, there was a modest 1.4-fold reduction in the initial rate of remodelling of acetylated H4 *versus* unmodified nucleosomes (Figure 6(c) and (d)). This raised the question of whether, as in the case of Isw2, the H4 tail was an allosteric activator of ATP hydrolysis or whether it was required as a binding epitope to recruit Chd1 to nucleosomes. Comparing the ATP hydrolysis rates of Chd1 with full-length and gH4 nucleosomes revealed that binding (*K*_m_) was unaffected (see also Supplementary Data, Figure 6(b)) and it was again the catalytic turnover rate which decreased in the absence of the H4 tail (Figure 6(e)).

### H4 acetylation leads to loss of histones *in trans*

Studies with the RSC and SWI/SNF complexes *in vitro* have shown that in addition to being able to redistribute nucleosomes along DNA fragments, they can transfer histone octamers from one DNA fragment to another at lower efficiency. This entails the displacement of the histone octamer from one molecule of DNA and subsequent transfer onto a separate one. The SWI/SNF complex has been implicated in the disassembly and subsequent reassembly of nucleosomes at the Pho5 promoter.^38,39^ This loss of nucleosomes at the promoter is correlated with spikes in histone acetylation levels, leading to the hypothesis that ATP-dependent remodellers may recognise this signal and promote transfer. To test whether there is a direct link between histone modification and octamer transfer, the efficiency of octamer transfer from different chromatin substrates by the RSC complex was measured.

**Figure 7:**
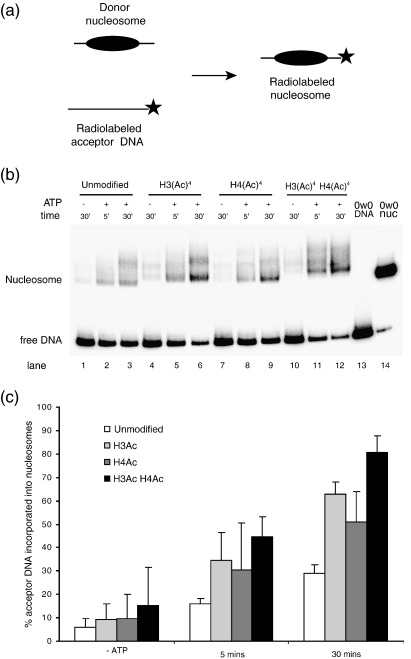
H4 tetra-acetylation increases octamer transfer by RSC. (a) Octamer transfer assay: RSC is able to disrupt nucleosomes and transfer the histone octamer from unlabelled donor nucleosomes onto a separate DNA molecule, in this case a radiolabelled 147 bp 0W0 fragment derived from the 601 positioning sequence. This is measured by the shift in mobility of a radiolabelled DNA fragment to that of a nucleosome. (b) Efficiency of octamer transfer from different donor nucleosomes. H3 tetra-acetylated nucleosomes are transferred faster than unmodified nucleosomes consisting of wild-type H3 and H4: compare lanes 4–6 with lanes 1–3. Surprisingly, H4 tetra-acetylated nucleosomes are also transferred faster than control: compare lanes 7–9 with lanes 1–3. When both H3 and H4 are acetylated the effect is additive (compare lanes 10–12 with 1–3). Lane 13 shows an equivalent amount of free DNA in the absence of RSC or nucleosomes and lane 14 is a nucleosome reconstituted separately on the same DNA fragment as a mobility reference. (c) Table plotting the amount of octamer transfer from different donor nucleosomes as the average of three independent repeats. Error bars represent the standard deviation. Although histones of wild-type sequence are used as a control in the data shown, octamers bearing cysteine mutations behaved similarly.

The octamer transfer reaction involves incubating a “cold” donor nucleosome with a “hot” ^32^P-labelled accepter DNA fragment in the presence of RSC and ATP. The reaction products were run on a native polyacrylamide gel and transfer is indicated by a shift in mobility of the “hot” accepter DNA. If the accepter DNA has a histone octamer transferred onto it, it should now have the same mobility as a control nucleosome reconstituted onto the same fragment (Figure 7(a)).

This was quantified as the fraction of radiolabelled free DNA incorporated into nucleosomes, as measured by mobility on a native polyacrylamide gel (Figure 7(b) and (c)). The amount of octamer transfer catalysed increased over time and was dependent on both ATP (Figure 7(b), lanes 1–3) and RSC (data not shown). As expected, octamer transfer from H3 acetylated nucleosomes was greater compared to unmodified nucleosomes (Figure 7(b), compare lanes 1–3 and 4–6). This is consistent with RSC having a lower *K*_m_ for H3 acetylated nucleosomes (Figure 4) and the preferential nucleosome sliding seen in Figure 3. Surprisingly, when we looked at H4 acetylated donor nucleosomes, these were transferred considerably more efficiently than unmodified nucleosomes. Indeed, the amount of transfer was almost as high as that of H3 acetylated nucleosomes (Figure 7(c)), despite RSC not showing any increased affinity towards H4 acetylated chromatin (Figure 4). A doubly H3 and H4 acetylated nucleosome was in turn transferred more efficiently than either of the single histone acetylations. This suggests that H4 acetylation does not act at the level of stimulating RSC directly but rather by predisposing the nucleosome to be remodelled *in trans*, perhaps by destabilising it. Consistent with this, we find that H4 acetylated nucleosomes are more prone to thermally induced histone H2A/H2B dimer loss than H3 acetylated nucleosomes (Supplementary Data, Figure 7).

## Discussion

### Histone acetylation alters the intrinsic dynamic properties of nucleosomes

Since they were discovered in the 1960's histone modifications have been predicted to affect chromatin structure and gene regulation.^40^ Subsequent studies have revealed correlations between histone modifications and transcription. For example, the recent application of genome-wide microarrays to map histone modifications, reveals that modifications such as H3 acetylation are found at the promoters of active genes and that their levels correlate with that of transcription.^41–46^ There are two prevailing views as to how modifications such as acetylation affect transcription. The first is based on the recruitment of activators due to recognition *via* modification binding modules. The number of modifications and respective binding domains identified has resulted in the hypothesis that these may form a code, which is read to determine a specific response.^47^ Alternatively it has been suggested that lysine acetylation may have a structural effect on chromatin structure by neutralising the charge interactions between histone lysine residues and DNA, resulting in a more open conformation.^48–50^ Several lines of evidence suggest that this can destabilise chromatin fibres.^19,51–53^ Histone acetylation may also affect the structure of mononucleosomes. Several studies suggest that DNA within acetylated nucleosomes is more accessible.^48,49,54^ It has been shown that acetylated histone tails adopt a more α-helical conformation,^55^ and that acetylated nucleosomes have a reduced linking number,^24^ consistent with wrapping less DNA. However, in this view, the role of individual histone modifications has been less clear. Here we show that acetylation of the H3 tail increases the inherent thermal mobility of nucleosomes whereas acetylation of H4 does not (Figure 2(e)). This increase in mobility does not appear to be due to charge neutralisation alone, as mutation of the relevant lysine residues to alanine did not produce the same effect (Figure 2(d)). The mechanisms behind this are not immediately clear; however, it is interesting to note that acetylation of H3 but not H4 increased the distance between the arms of linker DNA within a mononucleosome, as measured by FRET.^53^ We have also recently observed that individual histone tails have non-redundant effects on nucleosome dynamics,^27^ suggesting that the relationship between histone modification and chromatin dynamics may be more complex than initially appreciated. It will be interesting to examine how these effects interact with each other to alter chromatin structure and function *in vivo*.

### Histone acetylation affects ATP-dependent chromatin remodelling through altering either the *K*_m_ or *K*_cat_ of ATP hydrolysis

As their names suggest, ATP-dependent remodelling enzymes use the energy of ATP hydrolysis to alter chromatin structure. Based on the helicase domain of the catalytic subunit, chromatin remodelling complexes can be classified into a number of different subfamilies.^1^ Here we look at examples from three distinct subfamilies of chromatin remodellers: Chd1, Isw2 and RSC. The ATPase activity of these enzyme classes is stimulated by DNA and nucleosomes. Whereas it had been noted that histone tails and modifications affected their remodelling activity, it was not clear in many cases whether this was due to altered binding of modified nucleosomes or due to altered rates of ATP hydrolysis after binding. Using a real-time ATPase assay we show that depending on the particular enzyme involved either may be the case.

Analysis of the RSC complex confirmed that the improved remodelling of acetylated nucleosomes is due to higher substrate binding affinity and not due to increased ATP turnover (Figure 4(b)). This is consistent with previous observations of bromodomain containing remodelling enzymes, such as SWI/SNF, which show improved binding to acetylated chromatin templates.^12,56^ Remarkably, given the large number of bromodomains, the RSC complex shows no improved binding of H4 tetra-acetylated nucleosomes but rather, specifically H3 acetylated nucleosomes. By using chromatin with defined modification patterns we show that mono-acetylation at lysine 14 results in an increase in binding almost to the level seen with H3 tetra-acetylation. Consistent with this the Rsc4p subunit of RSC interacts genetically with H3 K14 but not H3 K9.^32^ Studies from the Workman laboratory have shown that SWI/SNF and RSC have increased affinity for nucleosomes that have been acetylated by the NuA4 HAT complex.^12,56^ We show here that RSC does not preferentially bind H4 K5,8,12,16 tetra-acetylated nucleosomes (Figure 4(b)); however, these observations can be reconciled by the fact that NuA4 shows HAT activity towards other histones besides H4, most notably H2A,^57,58^ and it may be this which results in the improved binding. For this reason, it would be interesting to test the affinity of RSC for nucleosomes containing acetylated H2A or H2B. Indeed, in high-resolution microarrays, H2A K7 acetylation but not H2B K16 acetylation is typically found in the same region of genes as H3 acetylation, and both are positively correlated with transcription.^46^

In the case of Isw2, we show that acetylation of H4 reduces nucleosome remodelling by lowering the *K*_cat_ of ATP hydrolysis and not by inhibiting nucleosome binding (Figure 5(c)). Thus, the unmodified H4 tail is required as an allosteric activator of ATP hydrolysis. This is consistent with the previous observation that acetylated H4 peptides have a reduced ability to stimulate ATP hydrolysis by dISWI in comparison to unmodified peptides.^34^ We also find that Chd1 activity is affected by modification and alteration to the H4 tail in a similar way to the Isw2 complex (Figure 6). It is notable that in the case of both Chd1 and Isw2, the effects of tail acetylation or truncation on ATPase activity are smaller than the effects on nucleosome sliding. It is possible that there is some amplification of the effect on ATPase activity due to multiple rounds of remodelling being required for repositioning. Alternatively, alterations to the H4 tail may reduce the efficiency with which these enzymes reposition nucleosomes in addition to altering their ability to hydrolyse ATP.

Previously, the requirement for the H4 tail for efficient nucleosome remodelling had only been observed for members of the Iswi subfamily of remodelling enzymes. However, there is evidence that there may be a significant degree of overlap in the function of Chd1 and Iswi subfamilies in *S. cerevisiae*. Isw1, Isw2 and Chd1 are all involved in transcription termination^59^ and their combined deletion is synthetic lethal with cellular stress.^60^ These complexes also reposition nucleosomes onto the same subset of thermodynamically favourable positions *in vitro*.^61^ Interestingly, like members of the Iswi subfamily, Chd1 is efficient in the generation of regularly spaced chromatin arrays,^62^ suggesting that a tightly coupled functional interaction with the H4 tail may be important for this activity. Studies with the similar *Drosophila* Mi-2 chromatin remodelling complex have shown that while its ATPase activity is stimulated by the assembly of histones into nucleosomes, this does not require histone tails.^63^ Whereas Mi-2 has similarity to Chd1, it is a member of a phylogenetically distinct group,^1^ suggesting that of the CHD-like remodellers, dependence on the H4 tail may be restricted to the Chd1 subfamily.

### H4 acetylation promotes octamer transfer *in trans* by the RSC complex

A distinct activity of the SWI/SNF and RSC complexes is their ability to remove nucleosomes and displace histone octamers *in trans*. The targeted removal of nucleosomes has been shown to be an important phenomenon in nuclear processes such as transcription^64–66^ and DNA repair^67,68^ and interestingly chromatin remodelling enzymes are involved in both.

In testing the role of histone modifications on octamer transfer *in trans* by RSC, we found that H3 tetra-acetylation resulted in a large increase in transfer (Figure 7(b) and (c)). This effect of H3 acetylation in promoting octamer transfer can be explained through improved recruitment due to the increased affinity for the modified nucleosome. Indeed, octamer transfer by the homologous SWI/SNF complex has been shown to require its bromodomains to mediate octamer transfer of SAGA acetylated nucleosomes.^69^ An unexpected finding was that H4 tetra-acetylation also had a stimulatory effect (Figure 7(b) and (c)), in stark contrast to the results seen with nucleosome repositioning *in cis* (Figure 3(b) and (d)). However, the additional effect of H4 acetylation was confirmed by the fact that a doubly H3 and H4 acetylated nucleosome was transferred better still than a singly H3 acetylated nucleosome. Kinetic analysis of ATPase activity clearly shows that H4 tetra-acetylated nucleosomes are not bound any better than unmodified nucleosomes and they do not alter the rate of ATP hydrolysis by RSC (Figure 4(b)). Thus, the increase in octamer transfer seen with H4 acetylated nucleosomes is not due to the modification recruiting or stimulating RSC activity but rather by affecting the nucleosome in such a way as to make it easier to transfer. In agreement with this, we find that H4 acetylated nucleosomes show increased thermally induced H2A/H2B dimer loss (Supplementary Data, Figure 7), indicating that this modification can affect chromatin dynamics independently of external factors. Interestingly, previous studies have shown that acetylation reduces the thermal stability of nucleosomes and H4 tetra-acetylation in particular reduces the thermal stability of nucleosomes almost to the level of bulk acetylation.^70,71^ The finding that histone acetylation improves the efficiency of histone octamer transfer by remodelling enzymes helps to explain discrepancies between previous studies of transfer efficiency. Previous studies that used native histones bearing modifications observed octamer transfer at a higher efficiency than was detected in another study using recombinant unmodified histones.^72^

The idea that acetylation of different histone tails may not be functionally equivalent is not a new one. Genome-wide microarray studies in yeast have shown that not all acetylation is positively correlated with transcription,^42,46^ and H4 acetylation does not substitute for H3 acetylation at Adr1-dependent genes.^73^ We propose that histone modifications may provide a means to facilitate histone eviction by ATP-dependent chromatin remodelling enzymes. Thus, acetylation, particularly of H4, may act in parallel with recruitment by activation domains to promote the removal of nucleosomes by remodelling enzymes such as RSC and SWI/SNF.^74^ This may also have relevance to processes other than transcription such as DNA repair. Intriguingly, H4 acetylation is very rapidly targeted to these regions^75,76^ making it tempting to speculate that this modification also acts to promote nucleosome loss during DNA repair.

## Experimental Procedures

### Purification of remodelling enzymes

TAP (tandem affinity purification) tagged yeast strains were either purchased from Euroscarf (Germany) for CHD1 and ISW2, or in the case of RSC the strain BCY211 was kindly supplied by Brad Cairns. Six litres of yeast were grown at 30 °C to an *A*_600_ of 2–2.5 in 3×yeast extract, peptone, adenine, d-glucose and frozen by dropwise addition into liquid nitrogen. Yeast cells were lysed by mechanical disruption using a blender (Waring) kept cold by addition of solid CO_2_. This was then thawed and purified using standard TAP protocols^77^ except using higher stringency wash buffers (20 mM Na-Hepes (pH 7.4), 350 mM NaCl, 10% (v/v) glycerol, 0.1% (v/v) Tween-20, 1 mM 4-(2-Aminoethyl) benzenesulfonyl fluoride hydrochloride, 2.6 mM aprotinin, 2 μg/ml leupeptin, 1 μM pepstatin). The purified eluate was concentrated using a Centricon YM-50 concentrator (Millipore) to 200–300 μl, dialysed against wash buffer without protease inhibitors and stored as 10 μl aliquots at −80 °C. The purity of Isw2 and Chd1 is indicated by Stockdale *et al.*,^61^ the purity of RSC complex is illustrated in Supplementary Data, Figure 8.

### Native peptide ligation

H3 Δ1-27 S28C (NCBI: CAD89679) and H4 Δ1-20 V21C (NCBI: CAD89677) were generated by sited directed mutagenesis. To ensure that the N-terminal cysteine within the globular histone was available for ligation, up to 10 mg of lyophilised histone was dissolved in 1 ml 6 M guanidine chloride(GnCl) (pH 7), 10 mM DTT, and incubated at 50 °C for 30 min. This was then dialysed extensively against three changes of 4l of 10 mM sodium acetate (pH 5.2), 1 mM EDTA, allowing at least 3 h per step. The fully reduced histone was then lyophilised and stored. Modified tail peptide thioesters were purchased from CSS-Albachem (Scotland) and aliquots of peptide in use stored at 10 mM in MilliQ water at −80 °C. The ligation conditions used were similar to those used by Dawson & Kent.^21,22^ The 1–2 mM globular histone was dissolved in 6 M GnCl, 0.2 M phosphate (pH 7.3) together with 0.4–0.5 mM thioester peptide and 2% (v/v) thiophenol, typically to a final volume of 200–300 μl. This was vortexed, and the reaction left at room temperature for 16–24 h. The reaction was stopped by adding DTT to 100 mM and dialysing the reactants against three changes of 500 ml SAUD0 buffer (7 M urea, 20 mM sodium acetate (pH 5.2), 5 mM 2-mercaptoethanol, 1 mM EDTA pH 8.0, 0 mM NaCl) using a 12–14 kDa MWCO dialysis membrane. The dialysed ligation mixture in SAUD0 buffer was spun to remove protein precipitate, and the supernatant loaded onto a 1 ml SOURCE 15S (Pharmacia) ion exchange column running at 1.5 ml/min to separate unligated globular histone from the full length product. A stepwise elution using SAUD0 (buffer A) and SAUD2000 (buffer B) (7 M urea, 20 mM sodium acetate (pH 5.2), 5 mM 2-mercaptoethanol, 1 mM EDTA (pH 8.0), 2 M NaCl) was used to elute in as small a volume as possible. The H3 Δ1-27 S28C and H4 Δ1-20 V21C constructs elute at a conductivity of 14 milliSiemens (mS), equivalent to approximately 180 mM NaCl, whereas full length histones elute at less than 25mS, 500 mM NaCl. This is typically 9.2%, and 25% SAUD2000, but due to variations in buffer preparation, the concentration of the two buffers required to obtain this conductivity should be determined empirically. We found that histones can elute in multiple peaks even though they are identical by SDS–PAGE and mass spectrometry, presumably representing differentially folded sub-species. The globular histone was eluted over 15 ml and the full-length histone over 10 ml, before the column was washed in SAUD2000 for 6 min in-between runs to remove tightly bound proteins and then re-equilibrated in buffer A. The relevant fractions are collected, diluted with SAUD0 to a final salt concentration below 100 mM NaCl and reloaded onto the column. We found that two to three rounds of purification were required to obtain greater than 95% pure ligated protein as determined by SDS–PAGE (Gradipore, Australia). The final fractions are concentrated using a YM-10 centricon (Millipore) to approximately 200 μl and their concentration measured by absorbance at 276 nm.

### Nucleosome reconstitution

Recombinant *Xenopus laevis* histone proteins were expressed and purified as described.^78^ Where necessary site-directed mutagenesis was carried out using the Stratagene Quickchange kit. Nucleosomes were assembled by mixing equimolar amounts of histone octamer and DNA in high salt and performing stepwise dialysis into low salt as described.^79^ DNA was generated by preparative PCR using primers obtained from Eurogentec (Belgium) fluorescently labelled where appropriate. Nucleosomes were assembled on DNA fragments based on the MMTV nucleosome A positioning sequence^80^ or the synthetically selected 601 sequence.^81^ We have adopted a nomenclature in which the lengths of DNA extensions on either side of a nucleosome are indicated as numbers on either side of a letter that defines the core positioning sequence used. So 54A18 designates a nucleosome positioned on the MMTV nuc A positioning sequence with a 54 bp extension to the upstream side and a 18 bp extension to the downstream side. The oligos used to generate the 54A18 fragment were 5′TATGTAAATGCTTATGTAAACCA and 5′TACATCTAGAAAAAGGAGC; for the 54A54 fragment 5′TATGTAAATGCTTATGTAAACCA and 5′ATCACATGTGAAAGTTAAAAAA; for the 0W0 fragment 5′CTGCAGAAGCTTGGTCCC and 5′ACAGGATGTATATATCTG; for the 54W0 fragment 5′TATGTCCATGCTCATGCC and 5′ACAGGATGTATATATCTG; for the 36W36 fragment 5′GGCGAATTCGAGCTCGGTAC and 5′AGGTCGACTCTAGAGAATCC. The PCR fragments were purified by ion exchange chromatography on a 1.8 ml SOURCE 15Q (Pharmacia) column. Radiolabelled DNA was prepared using T4 polynucleotide kinase (New England Biolabs) and [γ-^32^P]ATP (Molecular Bioproducts) according to the manufacturer's specifications.

### Nucleosome remodelling

Thermal remodelling reactions were performed by incubating nucleosomes in a thin-walled 200 μl PCR tube (ABgene, UK) in 50 mM NaCl, 50 mM Tris (pH 7.5) at 47 °C in a PCR machine with a heated lid for the specified amount of time. At the end of the reaction, sucrose was added to 5% (w/v) and the reactions placed on ice. The samples were separated on 0.2× Tris–Borate EDTA, 5% native polyacrylamide gels for 3.5 h at 300 V at 4 °C with pump recirculation. Gels were scanned in a Fuji Phosphoimager FLA-5100 and the bands quantified with Aida software (Fujifilm). For each time point the fraction of nucleosomes remodelled was calculated as the intensity of the destination position divided by the sum of the start and destination intensities. These data were then corrected to set the zero time point to zero percentage remodelled. The data points were plotted onto a graph and a hyperbolic curve of the form *y* = *x*/(*n+x*) fitted non-linearly to the data using the Solver add-in for Excel over 1000 iterative cycles. The initial rate of remodelling was calculated by differentiation of the curve and solving for time equals zero. At this point the initial rates for unmodified and modified nucleosomes within the competition assay were divided by each other to describe the initial rate of remodelling relative to the unmodified control. The average and standard deviations were calculated from at least three independent repeats. ATP-dependent remodelling reactions were performed as for thermal repositioning except using a reaction buffer containing 50 mM NaCl, 50 mM Tris (pH 7.5), 3 mM MgCl_2_, 1 mM ATP. These were incubated at 30 °C with the amount of remodelling enzyme and for the time specified in the figure legends. The reactions were stopped using 500 ng of HindIII-digested bacteriophage lambda competitor DNA, adding sucrose to 5% (w/v) and placed on ice. Nucleosome binding assays were performed in 50 mM Tris–HCl (pH 7.5), 50 mM NaCl, 3 mM MgCl_2_ and 3% (w/v) Ficoll-400 unless otherwise stated.

### ATPase assay

The ATPase assay was performed as described.^31^ The reaction was measured in solution in real-time on a Cary Eclipse fluorimeter (Varian, Australia). The excitation and emission wavelengths were set to 430 nm and 465 nm, respectively, with a 5 nm slit width and polarisers used to compensate for anisotropy. A calibration curve using MDCC–PBP at 5 nM and increasing amounts of inorganic phosphate was performed to determine the range across which the detection is linear. MDCC–PBP was diluted using 10 mM Pipes (pH 7) to 50 nM, this was mixed with nucleosome and enzyme in a final buffer concentration of 50 mM NaCl, 50 mM Tris (pH 7.5), 1 mM MgCl_2_ to a final concentration of 5 nM. Enzyme and nucleosome concentrations are as stated in the Figure legends. Measurements were performed with 1 mM ATP which had previously been treated to remove inorganic phosphate contamination.^31^ However, identical results were obtained using 100 μM ATP which had not been treated with the phosphate mop. The measurement of the rate of hydrolysis was performed using the Cary Eclipse software and non-linear fitting of the Michaelis–Menton equation to the data done within the Solver add-in for Excel over 1000 iterative cycles.

### Octamer transfer and dimer exchange

Octamer transfer was performed under the same conditions as standard ATP-dependent remodelling. The 15 nM donor nucleosome (assembled on unlabelled 0W0 DNA) was incubated with 10 nM RSC and 1 nM radiolabelled acceptor 0W0 DNA for the specified amount of time in the presence of 1 mM ATP. The reactions were stopped by the addition of excess cold DNA and analysed by native PAGE. Thermal dimer exchange was performed as described.^72^ Briefly, this is performed as for nucleosome repositioning, except with a twofold molar excess of H3/H4 tetrasomes assembled onto 147 bp ‘601’ DNA.^81^
