# Supplementary material for: Histone Modifications Influence the Action of Snf2 Family Remodelling Enzymes by Different Mechanisms
Source: J Mol Biol. 2007 Nov 30;374(3):563–79. doi: 10.1016/j.jmb.2007.09.059 (PMC2279226; doi:10.1016/j.jmb.2007.09.059)
Supplement: Supplementary material [file mmc1.pdf]

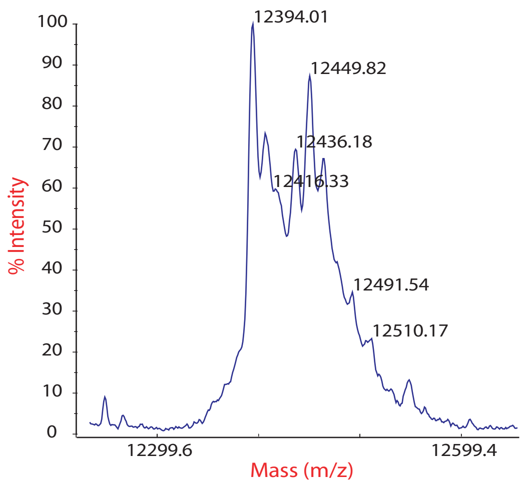

Supplementary figure 1. Efficient removal of initiator methionine

Mass spectrum of H3 ?1-27 S28C. The calculated mass of the protein without the initiator methionine is 12394Da and with the methionine is 12595Da. The peaks at 12416-12449Da represent low molecular weight adducts of the main peak at 12394 such as [M+Na]+ (12416 Da).

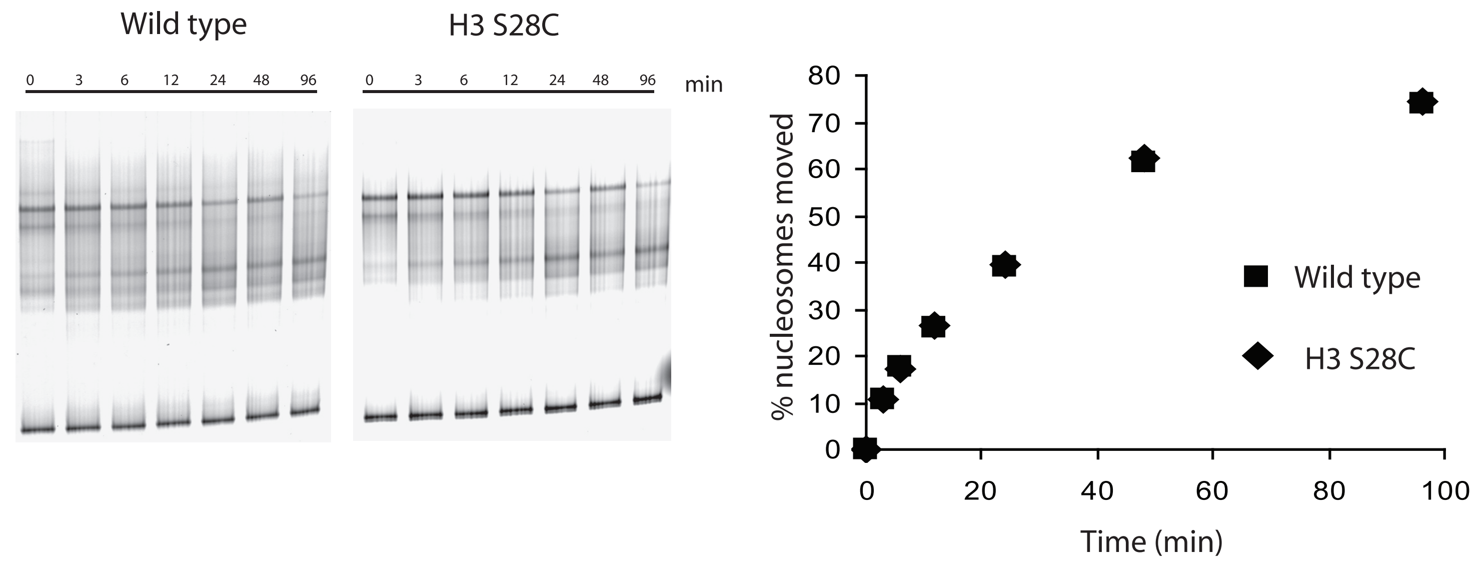

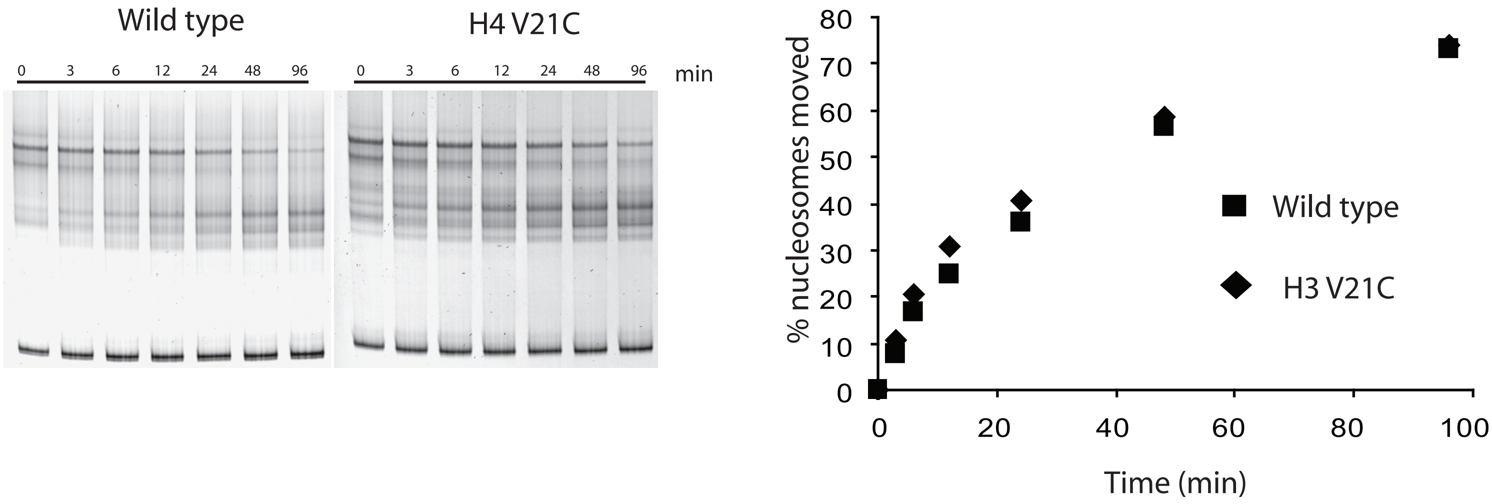

Supplementary Figure 2 - Cysteine point mutations have no effect on the thermal mobility of nucleosomes. Wild type nucleosomes assembled on cy5 labelled 54A54 DNA fragment were incubated at 47 °C as described for figure 2 togetherwith the indicated cystein point mutant nucleosomes assembled on Cy3 lablelled DNA. Scans of the two gels are shown along with a plot of the rate of nucleosome movement against time. It can be seen that the behaviour of wild type and cystein point mutatnt nucleosomes is indistinguishable.

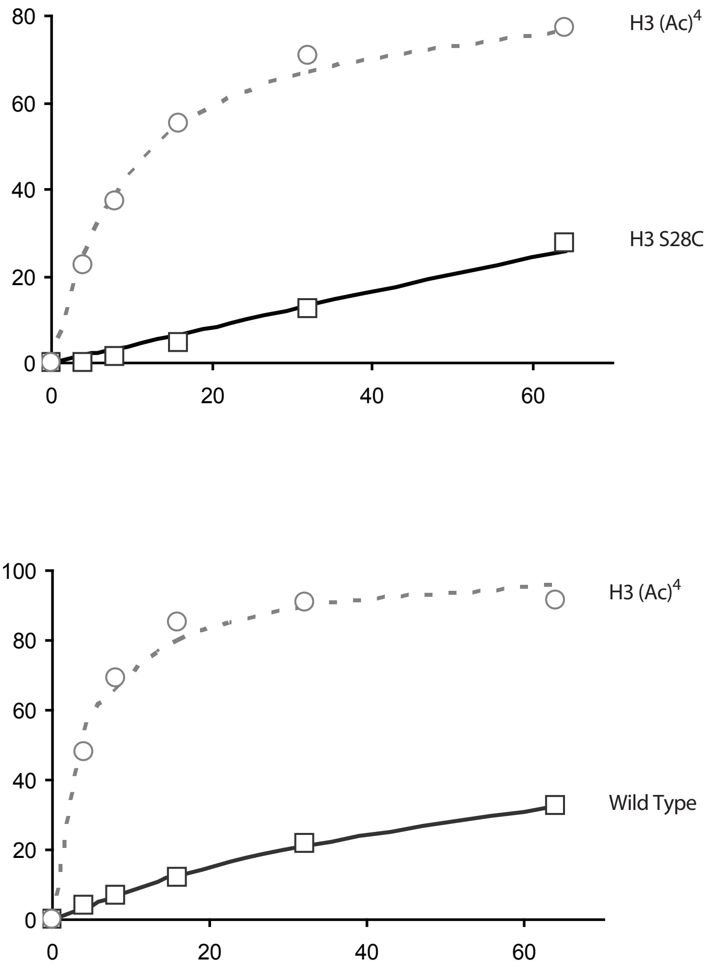

Supplementary Figure 3 Choice of control nucleosomes does not influence the effect of H3 acetylation. Graphs showing the effect of using the S28C mutation or wild type histones as a reference control for studying the extent to which RSC stimulates nucleosome sliding. Using S28C as a reference the initial rate of sliding of the H3 acetylated nucleosomes is 16 times faster. Using wild type H3 as a reference the initial rate of sliding of the H3 acetylated nucleosomes is 14.3 times faster. The difference between 16 and 14.3 is not statistically significant. This indicates that the choice of which histone to use as a control does not significantly influence the estimation of the effect of acetylation.

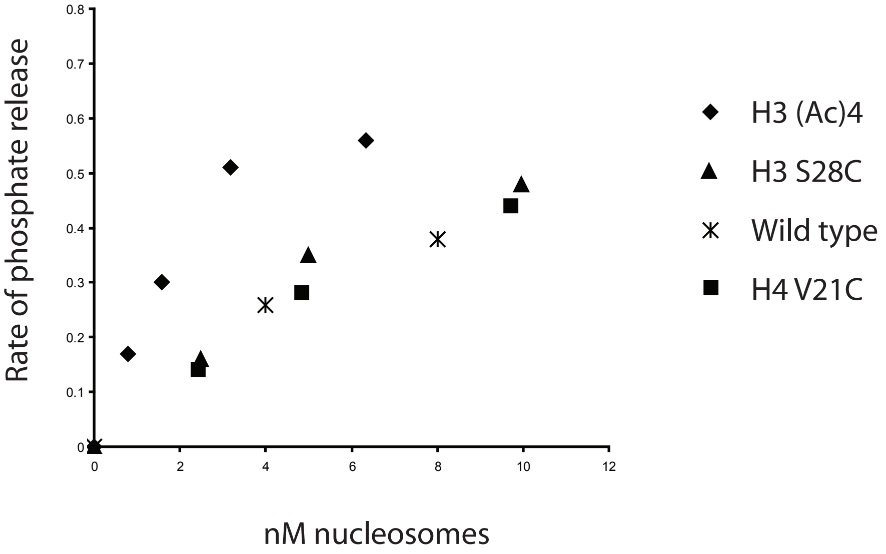

Supplementary figure 4 Stimulation of the ATPase activity of RSC in not affected by H3 S28C or H4 V21C mutations.

The figures show the rate of phosphate release for a range of concentrations of nucleosomes bearing different mutations. It can be seen that whereas H3 acetylated nucleosomes generate higher rates of ATP hydrolysis, the cysteine point mutations behave similarly to wild type nucleosomes.

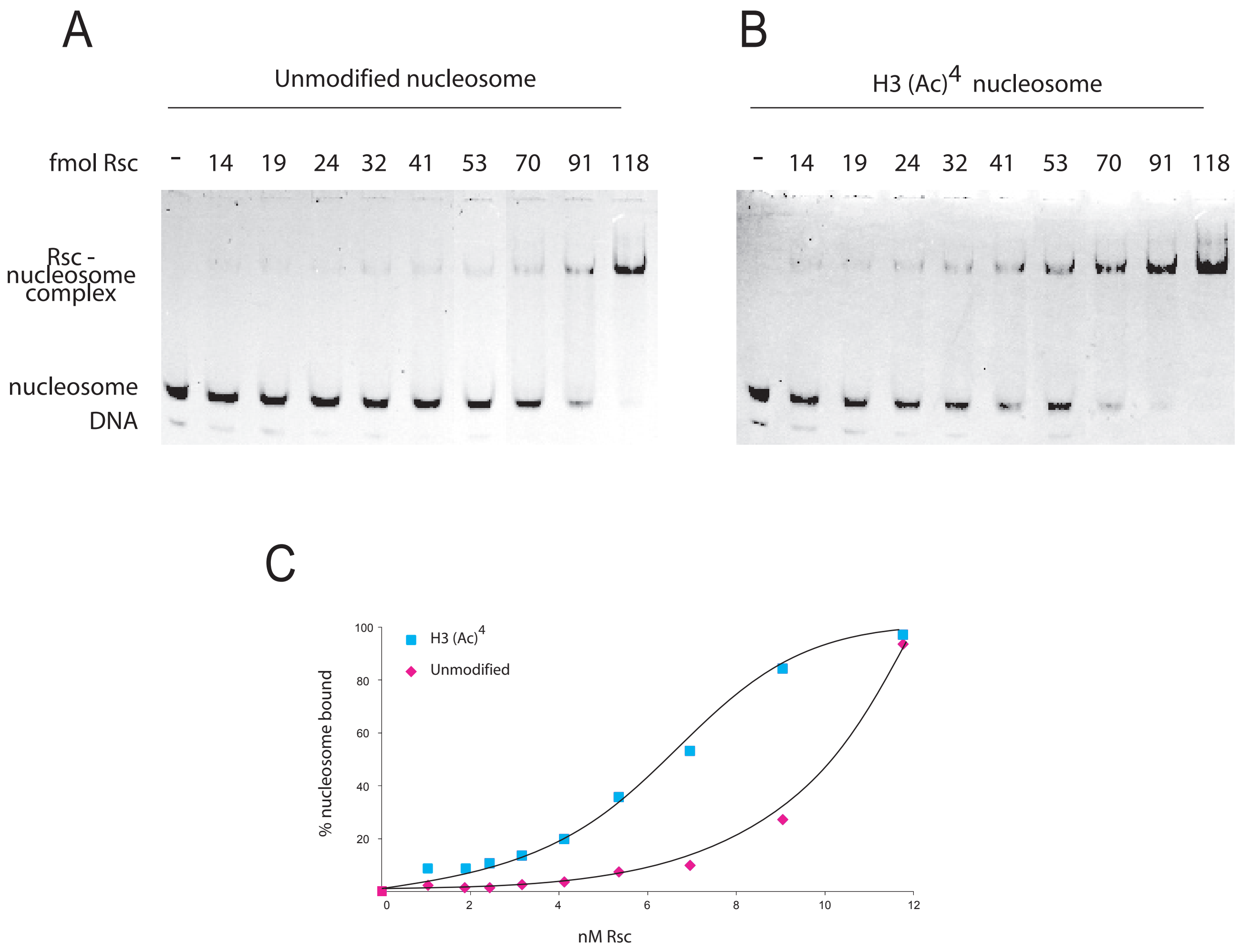

Supplementary figure 5. RSC shows higher affinity for H3 tetra-acetylated nucleosomes

1nM unmodified nuclesomes assembled on Cy3 labelled DNA and 1nM H3 tetra-acetylated nucleosomes assembled on Cy5 labelled 0W0 DNA were incubated with the indicated amounts of RSC. Scans of the gel to reveal the Cy3 signal corresponding to unmodified nucleosomes are shown in (A), and Cy5 signal corresponding to acetylated nucleosomes are indicated in (B). A graph showing quantitation is shown in (C).

A

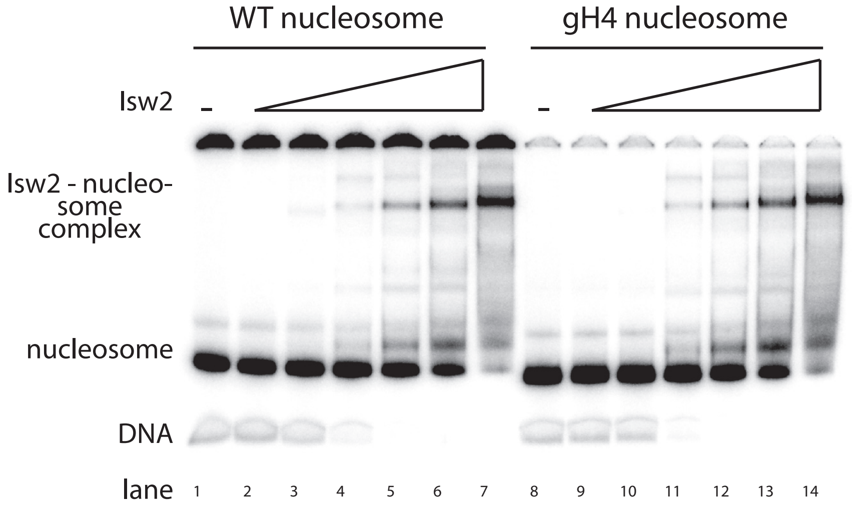

B

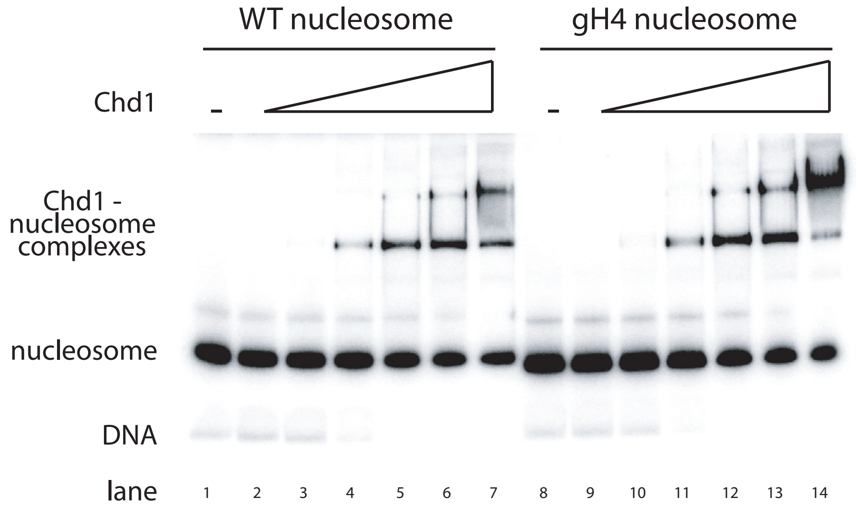

Supplementary figure 6. Isw2 and Chd1 do not require the H4 tail to bind nucleosomes

(A) 0.5pmol of either full-length or globular H4 radiolabelled 0W0 nucleosomes were incubated with increasing amounts of Isw2.
(B) 0.5pmol of either full-length or globular H4 radiolabelled 0W0 nucleosomes were incubated with increasing amounts of Chd1.

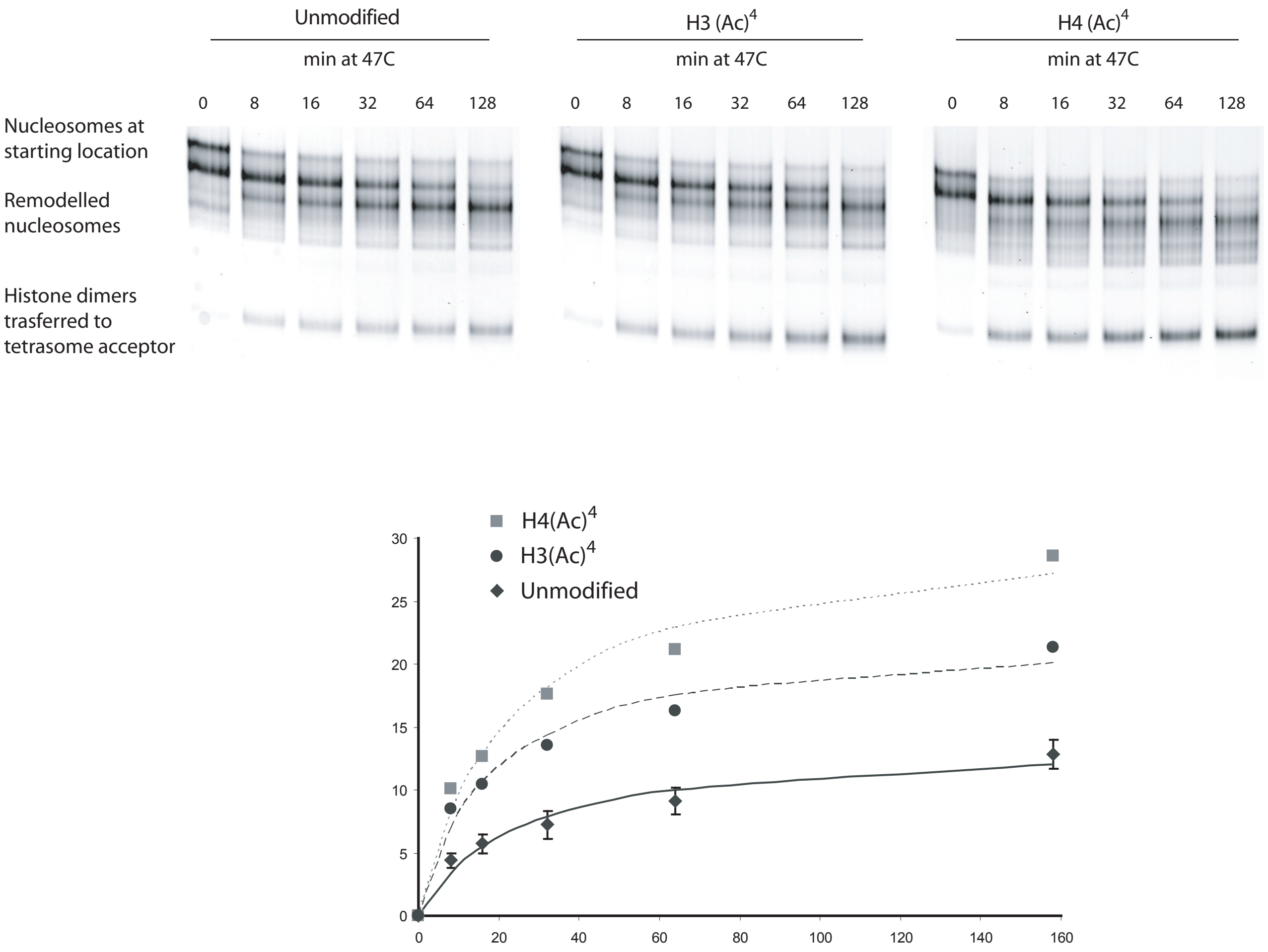

Supplementary figure 7. Thermally induced dimer exchange

(A) 2 pmol of 54A18 nucleosomes containing Cy5 labelled H2B were incubated at 47°C for the specified amount of time with 4pmol H3/H4 tetrasomes reconstituted onto 0W0. Transfer of the Cy5 signal to the faster migrating band indicates transfer of histone dimers from the nucleosome donor to the tetrasome acceptor.
(B) Quantification of the amount of dimer exchange from the different donor nucleosomes as a function of time. H4 acetylated nucleosomes are more prone to loss of dimers than either unmodified or H3 acetylated donor nucleosomes.

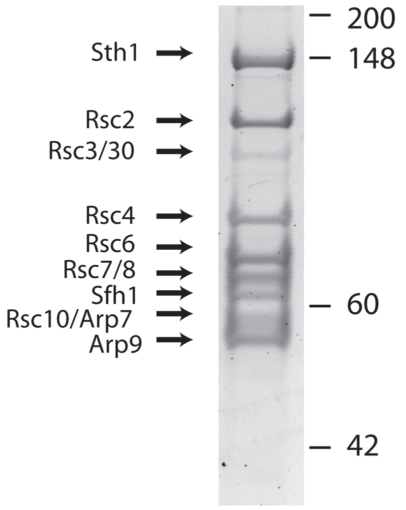

Supplementary Figure 8 Illustration of the purity of RSC complex used in this study
